# Hepatic hemangioma masquerading as a tumor originating from the stomach

**DOI:** 10.3892/ol.2015.2863

**Published:** 2015-01-12

**Authors:** XINGMAO ZHANG, ZHIXIANG ZHOU

**Affiliations:** Department of Gastrointestinal Surgery, Cancer Hospital, Chinese Academy of Medical Sciences, Peking Union Medical College, Beijing 100021, P.R. China

**Keywords:** hemangioma, liver tumor

## Abstract

Hemangioma is the most common benign hepatic neoplasm. The majority of cases are asymptomatic and can be confirmed by imaging examinations, including enhanced computed tomography and magnetic resonance imaging. Exophytic growth is not common and pedunculated cases are extremely rare. The present study reports a case that was pre-operatively misdiagnosed as a stomach-originating tumor. Laparoscopic exploration confirmed that this tumor was a hepatic hemangioma with a long peduncle originating from the left edge of the liver. The final diagnosis of cavernous hemangioma was confirmed by postoperative pathology. This indicates that hepatic hemangioma with a long peduncle has the possibility to be inaccurately diagnosed. Laparoscopic examination is required for such cases.

## Introduction

Hepatic cavernous hemangioma is the most common benign tumor of the liver and occurs more frequently in females (1). The majority of lesions are >3 cm in size and a significant proportion of patients are asymptomatic (2). Large lesions may cause a variety of symptoms, including an abdominal mass, pain, hemorrhage, jaundice, nausea and vomiting (3,4). Cavernous hemangioma usually presents as subcapsular, disclosed and solitary well-delineated nodules, these distinctive structures exhibit a characteristic hemodynamic pattern on enhanced computed tomography (CT) (5). Exophytic hemangioma, particularly in a pedunculated form, is extremely rare (6,7).

A patient possessing a pedunculated hepatic hemangioma that was misdiagnosed as a tumor originating from the stomach due to atypical imaging findings was recently treated at the Cancer Hospital (Chinese Academy of Medical Sciences, Peking Union Medical College, Beijing, China). In the present study, the case is reported together with a review of the literature. Written informed consent was obtained from the patient.

## Case report

A 52-year-old male was referred to the Cancer Hospital for ascending colon cancer, which had been confirmed by colonoscopy with biopsy. The patient had visited Chengde Central Hospital (Chengde, China) one week prior to attending the Cancer Hospital, and the chief complaint of the patient was blood-stained stool. Colonoscopy with biopsy was performed at the local hospital, and a lesion with a diameter of ~5 cm was identified in the ascending colon. The surface of the lesion was rough, and a diagnosis of ascending colon adenocarcinoma was confirmed by biopsy. No neoadjuvant therapy was delivered to the patient. With the aim of receiving laparoscopic surgery and better comprehensive treatment, the patient was referred to the Cancer Hospital, where further examinations were performed prior to surgery. The further examinations included enhanced abdominal and pelvic CT scanning; examination of tumor markers, liver and renal function and coagulation function; chest X-ray; abdominal ultrasound examination; and re-examination of the pathological results of the local hospital by two pathologists.

**Figure 1:**
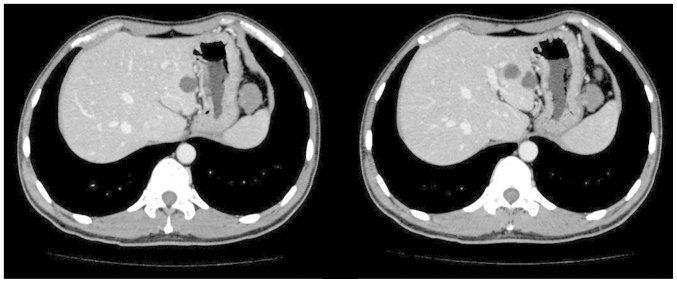
A mass with a maximum diameter of ~2.8 cm and a CT value of ~36 Hounsfield units was identified in the left upper quadrant (left). It appeared that the mass was originating from the body of the stomach (right), and a gastric stromal tumor was suspected due to the CT scans.

Enhanced CT revealed a mass in the left upper quadrant, with a maximum diameter of ~2.8 cm and a CT value of ~36 Hounsfield units (Fig. 1). This low-density mass was suspected to be a tumor originating from the stomach, as CT showed that the mass was adhered to the greater curvature of body of stomach and a peduncle appeared to originate from stomach wall. Hepatic ultrasonography did not reveal the mass.

The laboratory data were as follows: White blood cell count, 4,800/μl (normal range, 4,000–10,000/μl); red blood cell count, 5.3×10^12^/l (normal range, 3.5–5.5×10^12^/l); and hemoglobin, 82 g/μl (normal range, 120–160 g/μl). Tumor markers were as follows: α-fetoprotein, 3.2 ng/ml (normal levels, <20 ng/ml); carcinoembryonic antigen, 1.7 ng/ml (normal range, 0–5.0 ng/ml); and carbohydrate antigen 19–9, 16 U/ml (0–37 U/ml). Tests for hepatitis B surface antibody (HBsAb) provided a positive result, but the samples were negative for HBeAb, HBcAb, HB s antigen (Ag) and HBeAg; therefore, the patient in the present case did not have hepatitis B.

**Figure 2:**
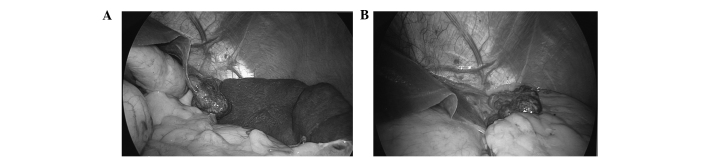
Laparoscopic exploration. (A) Dark red mass with a diameter of 3 cm, a smooth surface and a long peduncle was isolated from the liver extended down to the left side of the stomach. (B) The mass had a long peduncle originated from the left edge of the liver and was found in the left upper quadrant.

**Figure 3:**
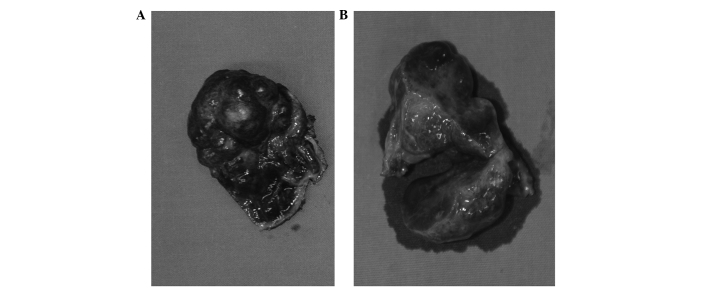
(A) The resected tumor was dark red in color with a complete capsule, soft texture and smooth surface; at its widest the diameter was ~3 cm. The diameter was ~3 cm. (B) On cutting the mass, the cross section was honeycomb-like, the inner surface was observed to be red and wet, and blood leaked from the section.

Based on preoperative examinations, including CT and blood tests, primary ascending colon cancer and cancer-related anemia were confirmed, and a left upper quadrant mass was also diagnosed. The patient was transfused with 800 ml blood and, two days after the transfusion, laparoscopic-assisted right hemicolectomy was performed. A dark red mass with a smooth surface and long peduncle originating from the left edge of the liver was identified in left upper quadrant. The mass, which had previously been suspected to be a tumor originating in the stomach, was confirmed as hepatic hemangioma by the findings of laparoscopic exploration. This tumor was isolated from the liver and extended down to the left side of the stomach (Fig. 2) and, macroscopically, the tumor size was 3.0×2.5×1.5 cm (Fig. 3). A resection of the hepatic hemangioma was successfully performed under laparoscopy.

For the ascending colon lesion, a diagnosis of stage T4aN0M0 adenocarcinoma was confirmed by postoperative pathology, and chemotherapy was recommended for this patient. This patient was administered chemotherapy four weeks following surgery. Oxaliplatin was administered intravenously in addition to an oral capecitabine regimen (oxaliplatin 235 mg over 2 h on day 1, capecitabine 3250 mg on days 1–14) for 2 consecutive weeks followed by 1 week of rest, for a total of 8 cycles (24 weeks). Four cycles had been completed when this report was started. During the follow-up period, examinations including clinical examination, abdominal ultrasonography, abdominal CT scanning, chest radiography and carcinoembryonic antigen assessment should be delivered for patients once every 3 months in the first 2 years after surgery, biannually in the next 3 years and then annually (8,9). For the hepatic hemangioma, a diagnosis of cavernous hemangioma was confirmed by pathology, and no other additional treatments were required for this hemangioma following surgery. At the time of writing, the patient had been followed-up for approximately three months following surgery.

## Discussion

Hemangioma is the most common benign tumor of the liver, and the incidence of hepatic cavernous hemangioma among liver tumors is as high as 20%. A diagnosis of typical hemangioma is straightforward when using a combination of various imaging techniques (6). Typical hemangioma presents early peripheral enhancement of the tumor on dynamic contrast CT, followed by centripetal fill-in of the contrast medium with persistently enhancement on delayed-phase images (10). In the present study, the tumor was revealed as low density in enhanced and delayed-phase images. Hemangioma is usually solitary and it is frequently in a subcapsular location, more commonly in the right lobe, particularly the posterior segment (1). Exophytic growth is not common and pedunculated cases are extremely rare (11–13). Hemangioma with a peduncle possesses the ability to isolate and migrate to the outside of the liver. For example, Moon *et al* (1) reported a case of pedunculated hepatic hemangioma that was misdiagnosed as a submucosal tumor of the stomach due to the atypical position of the tumor.

The majority of hemangiomas are asymptomatic, but larger hemangiomas can produce various symptoms, including an abdominal mass, pain, nausea, vomiting, jaundice, hemorrhage and even rupture. The patient in the present study did not experience any abdominal discomfort or an abdominal mass. No treatment is required for the majority of hemangiomas, with the exception of symptomatic treatment, which includes the treatment of a palpable mass, pain, increasing size or complications, including consumptive coagulopathy and rupture (14).

In conclusion, hepatic hemangioma with a long peduncle originating from the left edge of the liver may be inaccurately diagnosed. Laparoscopic examination is required for accurate diagnosis in such cases.
